# Spatiotemporal Dynamics of Early DNA Damage Response Proteins on Complex DNA Lesions

**DOI:** 10.1371/journal.pone.0057953

**Published:** 2013-02-26

**Authors:** Frank Tobias, Daniel Löb, Nicor Lengert, Marco Durante, Barbara Drossel, Gisela Taucher-Scholz, Burkhard Jakob

**Affiliations:** 1 GSI Helmholtzzentrum für Schwerionenforschung GmbH, Darmstadt, Germany; 2 TU Darmstadt, Institut für Festkörperphysik, Darmstadt, Germany; University of California-San Francisco, United States of America

## Abstract

The response of cells to ionizing radiation-induced DNA double-strand breaks (DSB) is determined by the activation of multiple pathways aimed at repairing the injury and maintaining genomic integrity. Densely ionizing radiation induces complex damage consisting of different types of DNA lesions in close proximity that are difficult to repair and may promote carcinogenesis. Little is known about the dynamic behavior of repair proteins on complex lesions. In this study we use live-cell imaging for the spatio-temporal characterization of early protein interactions at damage sites of increasing complexity. Beamline microscopy was used to image living cells expressing fluorescently-tagged proteins during and immediately after charged particle irradiation to reveal protein accumulation at damaged sites in real time. Information on the mobility and binding rates of the recruited proteins was obtained from fluorescence recovery after photobleaching (FRAP). Recruitment of the DNA damage sensor protein NBS1 accelerates with increasing lesion density and saturates at very high damage levels. FRAP measurements revealed two different binding modalities of NBS1 to damage sites and a direct impact of lesion complexity on the binding. Faster recruitment with increasing lesion complexity was also observed for the mediator MDC1, but mobility was limited at very high damage densities due to nuclear-wide binding. We constructed a minimal computer model of the initial response to DSB based on known protein interactions only. By fitting all measured data using the same set of parameters, we can reproduce the experimentally characterized steps of the DNA damage response over a wide range of damage densities. The model suggests that the influence of increasing lesion density accelerating NBS1 recruitment is only dependent on the different binding modes of NBS1, directly to DSB and to the surrounding chromatin via MDC1. This elucidates an impact of damage clustering on repair without the need of invoking extra processing steps.

## Introduction

When cells are exposed to ionizing radiation, DNA damage is induced. To ensure genomic integrity of the organism, cells respond in different ways to such DNA damaging events. The cellular repertoire of DNA damage responses includes the repair of the generated lesions, cell cycle arrest and apoptosis.

Double-strand breaks (DSBs), considered to be the most challenging type of DNA lesion, are first detected and stabilized by sensor protein complexes [1]. Subsequently, DSBs are repaired by distinct mechanisms, mainly homologous recombination (HR) and non-homologous end joining (NHEJ), reported to be error-free or error-prone, respectively [2]. These repair pathways involve a number of different proteins that bind to the damaged chromatin sites allowing the processing and ultimately the ligation of the broken DNA ends. Early activation steps in response to DSBs comprise post-translational modifications like the phosphorylation of the participating proteins at the break site or at the surrounding chromatin, thereby altering the modality of protein interactions. These modifications constitute key events in the regulation of repair and damage signaling pathways. In this study, we focus on dynamic events occurring early in response to DSBs.

Besides the KU/DNA-PK complex, which is known to be involved specifically in the process of NHEJ, the MRN complex is considered to be one of the first recognizing and binding to DSBs [3], [4]. It consists of the proteins MRE11, Rad50 and NBS1. Through Zinc hook interactions between Rad50 molecules, the DSB is stabilized [5]. Bound MRN then plays an important role in activating ATM, the key protein kinase in the mammalian DSB response [6]–[10]. However, some studies indicate that MRN is not absolutely required for ATM activation, but rather enhances its efficiency [11], [12]. During the activation process ATM autophosphorylates itself at various sites and transforms from inactive dimers to active monomers. These can subsequently phosphorylate the H2A histone variant H2AX in the chromatin surrounding the DSB [13]–[16]. Phosphorylated H2AX, referred to as γH2AX, forms a kind of loading platform for further proteins, e.g. MDC1 that binds to γH2AX and recruits more MRN and ATM leading to an amplification of the response [10], [17]–[21]. Using immunocytochemical staining techniques these protein accumulations and modifications at the DSB can be visualized as so called ionizing radiation-induced foci (IRIF) within the cell nucleus [22].

Many studies on DNA damage after ionizing radiation have been performed using fixed cells yielding information on repair protein hierarchies. However, these kinds of experiments are only snapshots of the protein distribution at a given time. Less is known about the dynamic behavior and binding characteristics of repair proteins in living cells. This is especially true in connection with the complex DNA damage that is induced after irradiation with densely ionizing charged particles. Low energetic ions deposit a high dose in a very small volume along the particle trajectory [23], [24] whereas the remaining part of the cell nucleus is practically spared [25]. As a result this localized dose deposition induces clustered DNA damage, consisting of multiple lesions in close proximity, along the ion track. The amount of energy transferred per particle track length is defined as LET (linear energy transfer) and serves as a measure of the density of damage produced. The aim of the present study was to describe the dynamic characteristics of early proteins in the cellular response to radiation-induced DNA damage, with emphasis on the impact of increasing lesion densities.

For this purpose we combined charged particle irradiation with live cell FRAP (Fluorescence recovery after photobleaching) and beamline microscopy techniques. Beamline microscopy allows measuring protein accumulation at DSBs in real time, whereas FRAP yields additional information on the diffusion, exchange and binding rate constants of these proteins. Using FRAP we present *in vivo* evidence for two different binding modes of NBS1 to sites of DNA damage. We show that the process of protein accumulation at DSBs depends not only on the protein itself but also on the damage density. With increasing LET the repair proteins NBS1 and MDC1, but not 53BP1, accumulate faster at DSBs. This is in line with recently reported data covering a lower LET range and showing faster recruitment of MDC1 but not 53BP1 for carbon ions compared to low LET protons [26].

To figure out the causal relations of the different protein in the interaction network, a mathematical kinetic model was established describing the observed experimental results. This model allowed us to identify the essential and thus most decisive reactions. On this basis we demonstrate that a shift in the contribution of the two NBS1 binding modalities leads to faster NBS1 accumulation at high LET. The model shows that the identical mechanism without extra processing steps or qualitative differences can explain the recruitment behavior at low and high LET values.

## Results

### Protein accumulation on damaged chromatin

The discrete sites of DNA damage produced by charged particle traversal provide a tool to dissect the immediate repair response in living cells. Here we want to explore whether the clustered damage and the potential interplay of different repair pathways at these sites of high lesion densities can influence the recruitment of early repair proteins. Therefore we used irradiation with different ion species to modulate lesion density and made use of the beamline microscope at GSI (GSI Helmholtz Centre for Heavy Ion Research) [27] to image protein accumulation in real time.

**Figure 1:**
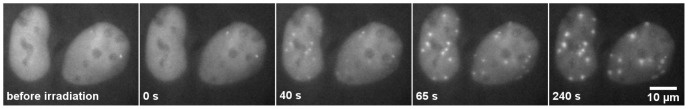
Beamline microscopy of U2OS cells expressing NBS1-GFP. Cells were irradiated with Sm ions (LET 10290 keV/µm) at 0 s generating DNA damage along their trajectory. These damaged sites are detected by the repair protein NBS1 and the amount of accumulated protein increases with time. This causes the formation of clearly visible foci and a rise in the fluorescent signal over time. Only selected time-points are shown.

**Figure 2:**
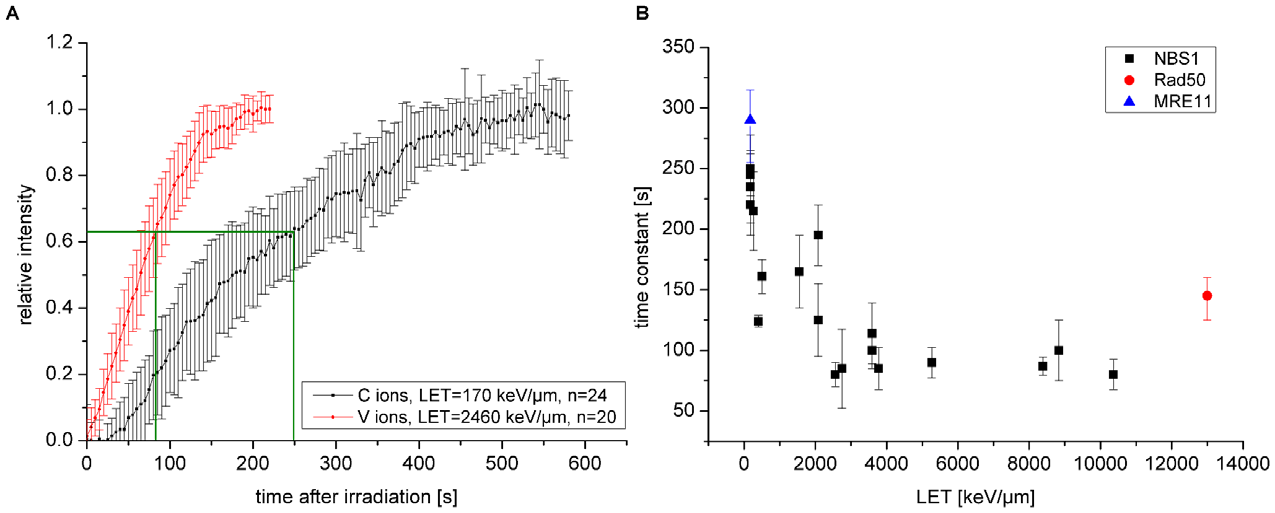
NBS1 protein accumulation at DSBs after ion irradiation. A: Normalized protein accumulation of NBS1 at DNA damage sites after C and V ion irradiation. When very high damage densities are created after exposure to a higher linear energy transfer (LET) radiation, NBS1 accumulates faster and saturates after shorter time. Error bars are 95% confidence interval. B: Monoexponential time constant, representing the time when 63% of the final foci intensity is reached (green lines in A), for NBS1 recruitment plotted as a function of the LET. Each LET value corresponds to one ion species. With increasing LET, the NBS1 accumulation accelerates up to about 3000 keV/µm and remains constant at further increasing ionization densities. Time constants of Rad50 and MRE11 accumulation are shown in red and blue respectively. Error bars are 95% confidence interval.

As exemplarily shown in Figure 1, images are taken before and immediately after irradiation to monitor the IRIF formation of fluorescently-tagged repair proteins. The fluorescence intensity in the foci increases until it saturates into a steady state. To quantify the kinetics, we plotted the normalized mean fluorescence intensity in IRIF as function of time (e.g., Figure 2 A).

The accumulation of the early repair protein NBS1 is shown after C- and V-ion irradiation, with an LET of 170 keV/µm and 2460 keV/µm, respectively, in Figure 2A. Additional data sets for the LET range from 170 keV/µm to 10290 keV/µm are shown in Figure S1. Irradiation with higher LET ions, corresponding to a higher damage density, leads to an increased (not shown) and more importantly also faster NBS1 protein accumulation, clearly visible after normalization of the plateau value to 1. Besides some small deviation in the initial slope, the course of fast protein accumulation can be described in good approximation by a mono-exponential curve characterized by a single time constant. Therefore, we evaluated this time constant representing the time after irradiation when ∼63% of the final intensity is reached. Figure 2B shows the time constant determined in this way for NBS1 recruitment as a function of the LET. Each LET value corresponds to a different ion species. With increasing LET, the time constant decreased or accordingly the recruitment accelerated up to an LET of about 3000 keV/µm and then stayed constant for higher LETs indicating a non-linear relationship. The corresponding time constants for Rad50 and MRE11, the other members of the MRN complex, are also included in Figure 2B after Au- and C-ion irradiation, respectively. Both values are comparable to that of NBS1.

**Figure 3:**
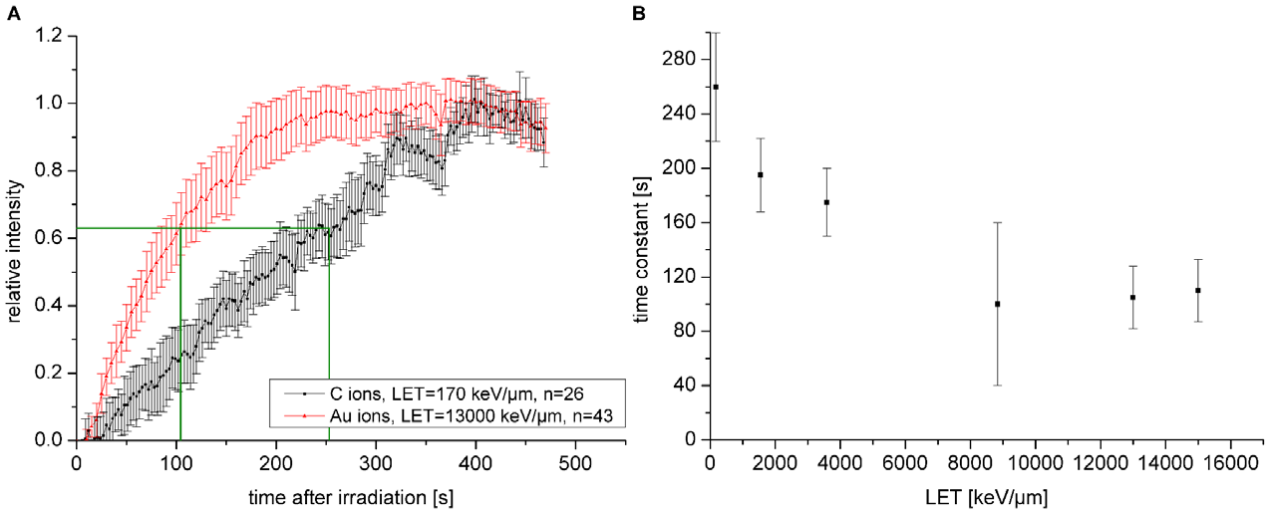
MDC1 protein accumulation at ion tracks. A: Normalized MDC1 protein recruitment to DNA damage sites after C- and Au-particle irradiation. Like NBS1, MDC1 accumulates faster at very high damage densities. Error bars are 95 % confidence interval. B: Monoexponential time constant, representing the time when 63% of the final foci intensity is reached (green lines in A), for MDC1 accumulation plotted as function of the LET. MDC1 protein accumulation accelerates with increasing LET, but saturates at higher LET values above 9000 keV/µm. Error bars are 95% confidence interval.

After ATM is activated via bound NBS1 [6]–[10] it phosphorylates H2AX in the chromatin surrounding the DSB [16], [28]. MDC1 can bind to γH2AX and accumulates in the vicinity of the DSB [17], [18], [20] forming a loading platform for more MRN molecules that leads to a signal amplification. Therefore, to study these binding processes downstream of ATM activation we measured the kinetics of MDC1 protein accumulation after irradiation with charged particles as shown in Figure 3. Consistent with the expectation, MDC1 recruitment occurred on a very similar timescale compared to NBS1 and, also similar to NBS1, MDC1 accumulated faster after irradiation with particles of very high LET. For an LET higher than 9000 keV/µm there was no additional acceleration in the recruitment kinetics.

To check whether this altered protein accumulation kinetics with increasing LET is a general phenomenon of repair factors, we analyzed the protein 53BP1 which only binds to the chromatin surrounding the DNA damage. To cover the wide range of LET, 53BP1 recruitment was measured after C-and Au-ion irradiation (LET of 170 keV/µm and 13000 keV/µm, respectively) and found to be almost identical (Figure S2). Even a very high lesion density did not influence the kinetics of 53BP1 recruitment, indicating that it is independent of LET. Apart from that, the 53BP1 accumulation kinetics revealed a pronounced lag phase and was clearly slower compared to NBS1, ATM and MDC1. The fact that not all repair proteins exhibit the LET-dependent recruitment behavior of NBS1 or MDC1 supports the notion that the accelerated accumulation has a mechanistic basis.

We previously published the kinetics of ATM accumulation after charged particle irradiation measured by beamline microscope [13]. The results yield a recruitment of ATM with a time constant of ∼125 s for U ions (LET 14350 keV/µm), also consistent with the early role of ATM in the phosphorylation of H2AX.

### Mobility and binding characteristics of proteins

#### Protein mobility in untreated cells

The accumulation kinetics is determined by the mobility of the proteins, the velocity of binding site generation, and the binding characteristics of the protein. To discriminate between these factors, we first analyzed the general protein mobility in the nucleus of untreated cells using FRAP. Furthermore, the previous evaluation of the general mobility of the protein of interest is a prerequisite for the analysis of protein binding after irradiation. During the FRAP measurements, the fluorescent tags of the protein were irreversibly bleached in a small region inside the cell nucleus. The exchange of bleached proteins with the surrounding unbleached ones, becomes visible by the signal recovery and directly reflects protein mobility. To gain insight into the mobility of a non-interacting protein in the nuclear environment pure recombinant GFP was expressed. The resulting curve for GFP mobility in the nucleus of living human U2OS cells is shown in Figure S3. The FRAP curve can be reproduced with a mathematical diffusion model according to Soumpasis [29]. We obtained a diffusion coefficient D  =  D_eff_ for pure GFP of 12 µm^2^/s in the U2OS cell nuclei.

Assuming spherically shaped proteins and equal protein density, we could estimate the diffusion coefficient of a GFP tagged protein based on the mass ratio between the GFP-tagged protein and pure GFP and on the diffusion coefficient of pure GFP [30]. Diffusion coefficients D_calc_ resulting from this calculation are shown for NBS1 and MDC1 in Table 1.

**Table 1:** Theoretical free diffusion constants D_calc_ for GFP-tagged NBS1 and MDC1 proteins estimated from the diffusion constant for GFP and the mass difference between pure GFP and the tagged proteins as well as experimental effective diffusion constants D_eff_ from experimental FRAP curves.* For free GFP D_calc_  =  D_eff_.

	GFP	NBS1-GFP-GFP	MDC1-GFP
mass [kDa]	27	137	257
D_calc_ [µm^2^/s]	12*	7.0	5.7
D_eff_ [µm^2^/s]	12	0.25	0.029

**Figure 4:**
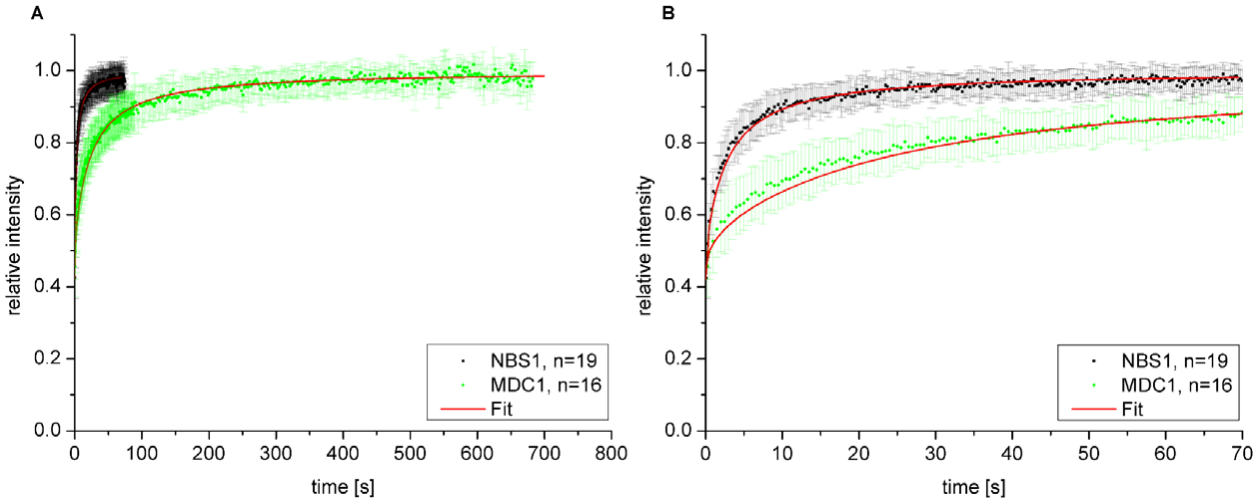
Repair protein mobility in untreated cells. A: FRAP curves of GFP-tagged NBS1 and MDC1 in untreated U2OS cells. Error bars are standard deviation. B: Enlarged section from A from 0 s to 70 s. Effective diffusion fits are shown in red. Error bars are standard deviation.

D_eff_ values were measured by analyzing the mobility of the GFP-tagged repair proteins NBS1 and MDC1 in untreated cells using FRAP and subsequent fitting of the data with the mathematical diffusion model as applied before [29], [31]. The measured fluorescence intensity as a function of time is shown in Figure 4. The mobility of MDC1 was found to be considerably slower than that of NBS1. The fits of the mathematical diffusion model to the data obtained for NBS1 and MDC1, which are the basis of the values of D_eff_ shown in Table 1, are also shown in Figure 4. Whereas the overall fits describe the data reasonably well, there are small deviations for the MDC1 data at short times. However these measured D_eff_ values are much smaller than the derived D_calc_ values based on the mass ratio calculations (compare 2^nd^ and 3^rd^ row in Table 1). The generally lower value of the measured effective diffusion constant D_eff_ of NBS1 and MDC1 may be due to a temporary binding of these proteins through transient interactions.

According to the measured effective diffusion constants, it takes on average 0.83 s for a GFP protein to travel a distance of 6.3 µm. In a cell of cylindrical geometry with a radius of 9.4 µm, this corresponds to the average distance to the center of a cell nucleus for any molecule. For the NBS1 and MDC1 protein it takes ∼40 s or even ∼340 s, respectively.

#### Experimental setup for determining the binding behavior on damaged chromatin

**Figure 5:**
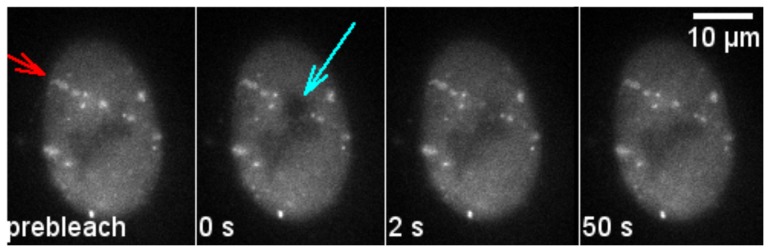
FRAP measurement of repair proteins bound at damaged DNA. U2OS cells expressing NBS1-GFP were irradiated with Ti ions (LET ∼270 keV/µm) under a low angle resulting in a streak-shaped foci pattern along the ion trajectory (red arrow). At time 0 s the fluorescence tag of the proteins in a small part of the streak are bleached with a short and intense laser pulse (cyan arrow). Fluorescence recovery in the bleached region represents the protein exchange at the DNA damage. Selected time frames are shown. Time labels correspond to the time after bleaching.

To elucidate the protein binding behavior at radiation-induced DNA damage sites, we irradiated cells with charged particles under a low angle resulting in a streak-shaped IRIF pattern [32]. This allowed discriminating between foci induced by irradiation and spontaneous foci occurring in untreated cells. The fluorescence signal of the accumulated proteins was bleached within single IRIF along the track. Fluorescence recovery was monitored over time (Figure 5). The resulting curve also reflects protein binding at radiation-induced DNA damage.

#### Binding of NBS1 at damaged chromatin

**Figure 6:**
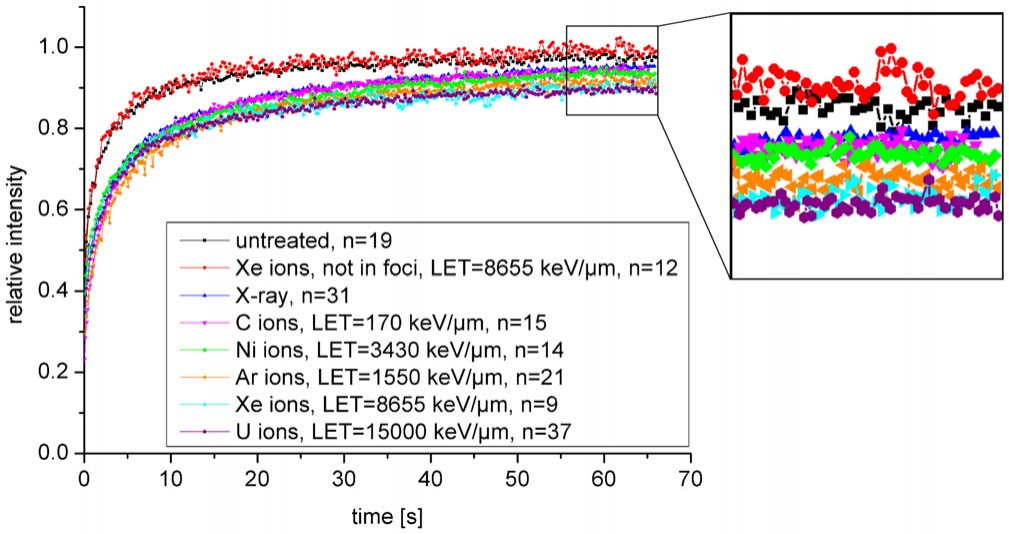
FRAP measurements of NBS1 binding on damaged DNA after irradiation with ions of different LETs. NBS1 proteins bound to damaged DNA showed a reduced mobility compared to unbound proteins. For times beyond ∼10 s the FRAP curves showed a shallower increase with increasing LET. Error bars are not shown for the sake of clarity. Exemplary error bars are included in Figure 4 and Figure 7, the others are comparable.

Aimed at obtaining a more mechanistic insight into the observed LET dependence of NBS1 accumulation, we analyzed NBS1 binding at DSBs using FRAP measurements (Figure 6). The recovery curves of NBS1 in non-irradiated cells and in irradiated cells in regions outside of IRIF were identical, indicating that the effective diffusion of the NBS1 in the nucleus was not affected by the irradiation. In contrast, the mobility inside radiation-induced foci was clearly reduced. With increasing LET, i.e., increasing damage density, the FRAP curves showed a shallower increase at longer times (Figure 6 insert). This indicates that not only the recruitment kinetics, but also the binding constants change with the LET.

**Figure 7:**
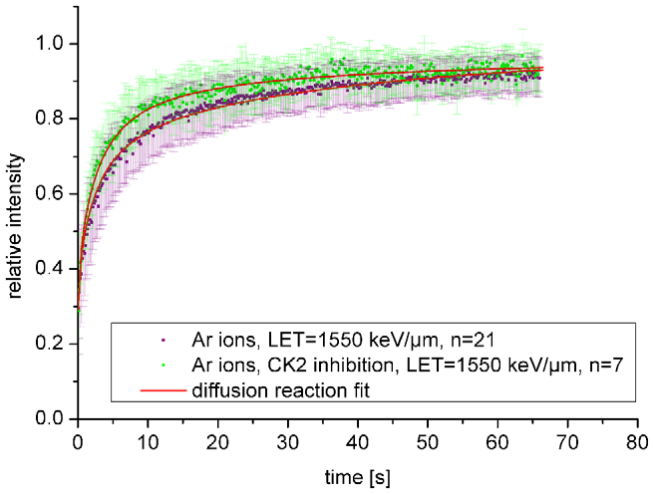
NBS1 binding at damaged DNA following CK2 inhibition. NBS1 binding at damaged DNA after CK2 inhibition preventing the interaction between NBS1 and MDC1. Cells were irradiated with Ar-ions. Error bars are standard deviation.

**Figure 8:**
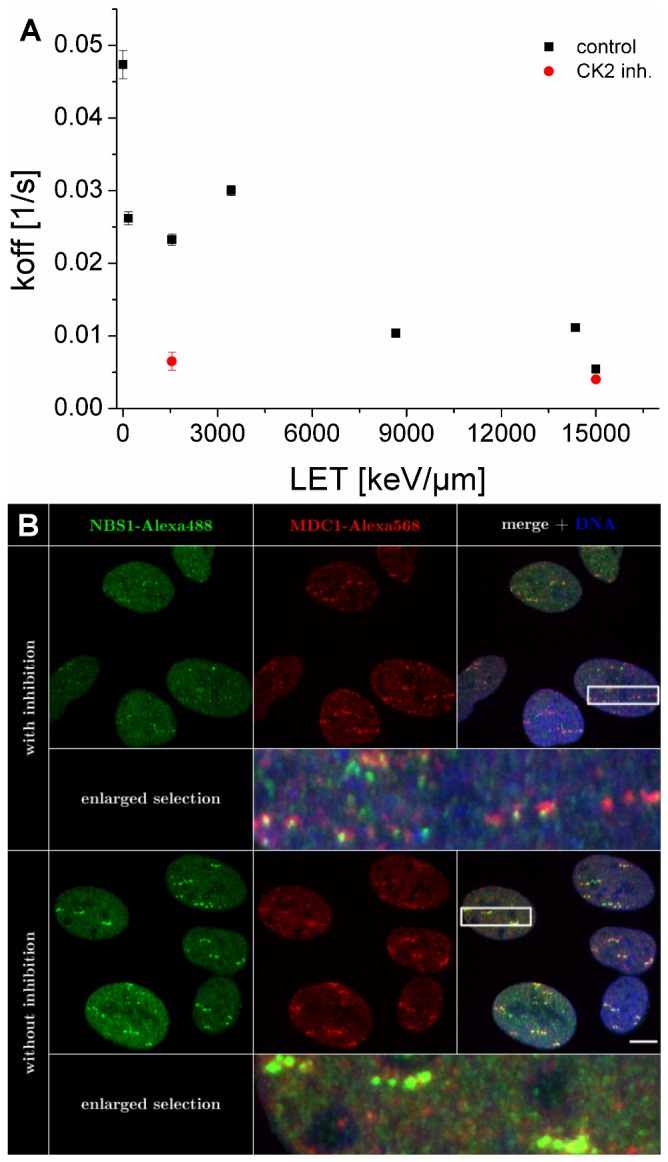
Influence of LET and CK2 inhibition on NBS1 binding to IRIF. **A) NBS1 dissociation constant koff versus the LET.** Values were obtained by fitting the FRAP curves with the diffusion reaction model described by Sprague and coworkers [40]. As the LET increases, protein binding consta*n*ts approach the values of NBS1 binding obtained with CK2 inhibition. Error bars correspond to the asymptotic standard error. **B**) **Influence of CK2 inhibition on NBS1 and MDC1 foci size.** Immunofluorescence staining of NBS1 and MDC1 after Au ion irradiation with and without CK2-inhibition. U2OS cells were fixed 10 min after Au ion irradiation and immunocytochemically stained against NBS1 (green) and MDC1 (red). DNA was counterstained with DAPI (blue). Scalebar 10 µm.

As described earlier, NBS1 as part of the MRN complex is considered to bind to the radiation-induced focus in two different ways: either more directly on DSB ends and/or associated to the surrounding chromatin via MDC1. Aimed at distinguishing these two fractions and to measure the binding constant of the direct binding to DSB ends only, we inhibited the casein kinase 2 (CK2) using 4,5,6,7-tetrabromotriazole (TBB) and irradiated with intermediate LET (1550 keV/µm) argon (Figure 7, 8A) or high LET (15000 keV/µm uranium ions, Figure 8A). CK2-dependent phosphorylation of MDC1 occurs independent of irradiation, but is a prerequisite for NBS1 binding to MDC1 after introducing DSBs [21], [33]–[35]. Under conditions of CK2 inhibition, using immunocytochemical staining we detected smaller, so called microfoci [36] of NBS1 embedded in the MDC1 signal as shown here (Figure 8B) for gold ions with a high LET (13000 keV/µm) similar to uranium. Obviously, even at this high LET, a significant contribution of the outer focus to the NBS1 IRIF can be seen which is abolished by CK2 inhibition. It must be noted that these high LET-induced microfoci do not represent single DSBs, but might correspond to local acting repair centers gathering multiple DSBs [37], [38] and also reflect the local spatial saturation of the number of the number of IRIF after high LET [39].

The FRAP curves, especially at the lower LET showed a steeper increase for short times and an earlier saturation behavior (Figure 7). To extract association and dissociation rate constants from these types of FRAP measurements we applied the mathematical radial diffusion-reaction model described by Sprague [40]. As it is based on additional binding sites in a small volume within a cell nucleus it matches our circumstances. The limitations of the model have been previously described in detail [41], [42]. For simplification we used the radial 2 dimensional FRAP model, not considering 3D effects and assuming a cylindrically shaped cell nucleus.

Fitting results are exemplary shown for the argon experiment in Figure 7. Obviously, the data could be well represented by this model. By quantifying the effective diffusion behavior outside the IRIF as described before, this procedure allowed determining the effective association- (k*_on_) and dissociation (k_off_) constants at damaged chromatin. For all FRAP curves these values were determined (Table S1). The effective association constants k*_on_ are defined as the product of the free binding site density in equilibrium and the actual association constant k_on_. Note that the free binding site density and thus k_on_ are not directly accessible. However, k_off_ values do primarily reflect NBS1 binding characteristics and the results are plotted in Figure 8A in dependence of the LET.

With increasing LET, i.e., increasing lesion density, the binding constants decrease and approach values close to the one obtained after lower LET irradiation but with CK2 inhibition. Remarkably CK2 inhibition had only a minor effect at high LET values (uranium). Both observations suggest that the proportion of NBS1 bound in the inner focus (i.e., more directly at the DSBs) increases with increasing LET.

#### Binding behavior of MDC1 on damaged chromatin

**Figure 9:**
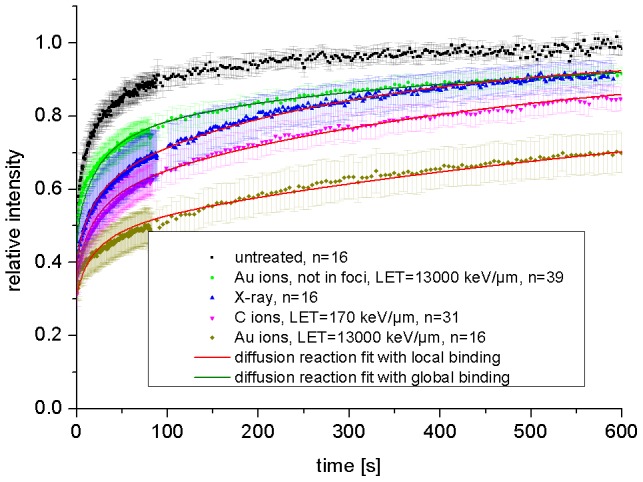
FRAP curves of MDC1 binding after charged particle irradiation. The mobility of MDC1 is drastically reduced at damaged DNA. MDC1 mobility is not only reduced at damaged sites but also in the whole nucleus when very high damaged densities are generated after heavy charged particle irradiation. Error bars are 95% confidence interval.

To study the binding of MDC1 at DSBs and its LET dependent behavior in comparison to NBS1, we performed FRAP experiments with MDC1-GFP after irradiation. The resulting curves are shown in Figure 9. The radiation types were selected to span the entire LET range and IRIF were bleached after low LET X-rays, 170 keV/µm C- and 13000 keV/µm Au ion irradiation. To analyze the mobility of MDC1 proteins not bound to DSBs, an arbitrary region within an Au-ion irradiated nucleus but outside the DNA damage streak was bleached (referred to as “not in foci”). The FRAP curve of non-irradiated (untreated) cells is shown for comparison.

In general the turnover of MDC1 bound in foci to damaged DNA was much slower compared to that of NBS1 (compare Figure 6 and Figure 9). Unexpectedly, after very high-LET irradiation MDC1 showed a strong reduction in mobility not only in close proximity to DNA damage (in foci) but also over the whole cell nucleus compared to unirradiated cells (compare the green and black dots in Figure 9).

Fitting the “not in foci” FRAP curve after Au ion irradiation with the diffusion model as applied to the untreated cells did not give satisfactory results, confirming additional binding (not shown). Therefore, we described this pan-nuclear MDC1 binding after high-LET irradiation also with a diffusion-reaction model. In this model, however, the additional binding sites are homogeneously distributed in the whole nucleus. It is referred to as diffusion reaction model with global binding [43]. This model describes the data reasonably well (green line in Figure 9) yielding effective association and dissociation constants for pan-nuclear MDC1 binding of: k*_on_ = (74±2)·10^−5^ 1/s and k_off_ = (193±4)·10^−5^ 1/s.

For the binding of MDC1 in radiation-induced foci we applied the diffusion reaction model with local binding as before. This model describes binding sites located in a small sub-volume (foci) inside the cell nucleus [40]. The corresponding fit curves are shown in red in Figure 9. For MDC1 binding at DSBs after X-ray irradiation we determined effective association and dissociation constants of: k*_on_ = (587±13)·10^−3^ 1/s and k_off_ = (425±6)·10^−5^ 1/s. This model with local binding assumes an effective diffusion behavior of the unbound protein fraction. Thus, after high-LET irradiation where pan-nuclear MDC1 binding is observed, this model does not adequately describe the situation. Accordingly the model fit showed some small deviations from the experimental data after C- and Au-ion irradiation and the corresponding binding constants could not be obtained precisely. Nevertheless the obtained values for k*_on_ and k_off_ were of the same order of magnitude, with differences of less than a factor of 2 compared to X-ray irradiation The LET dependent shift of the FRAP curves is due to an increase of free MDC1 binding sites in the equilibrium. This can be caused by more binding sites as well as less free MDC1 in the bleached spot due to nuclear-wide binding after high LET irradiation. Compared to the binding in IRIF the k_off_ value for pan-nuclear MDC1 binding was in the same range, whereas the k*_on_ value was drastically reduced, indicating a similar binding mode but a strongly reduced density of free binding sites.

### Numerical model

**Figure 10:**
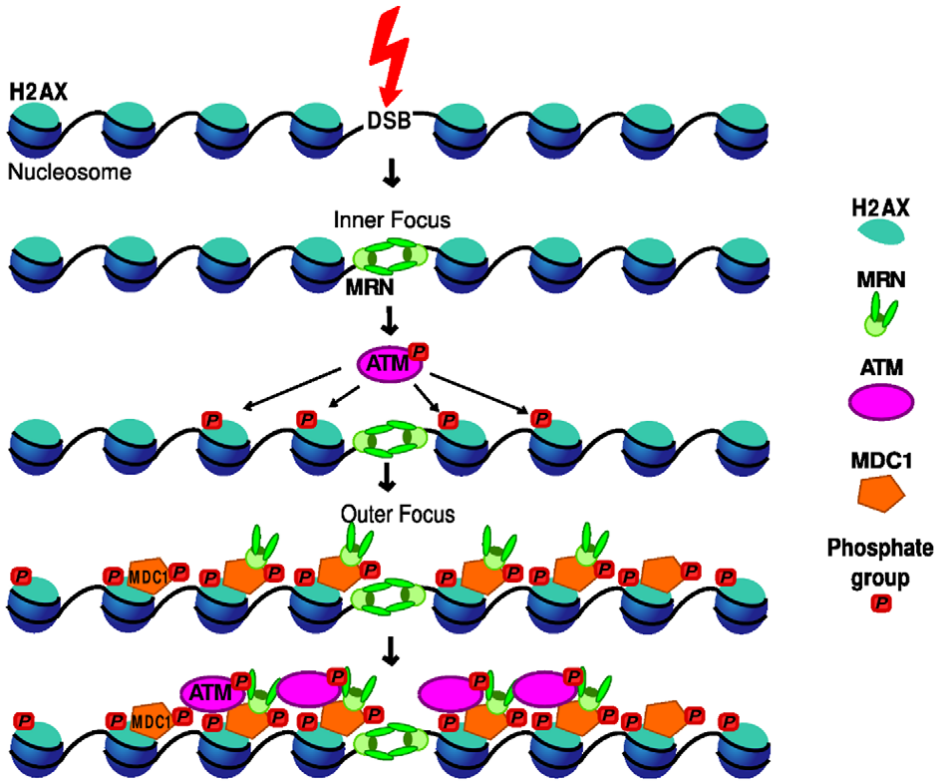
Schematic of interactions in our minimal model. MRN binds directly to the DSB strand ends. ATM is activated there and subsequently phosphorylates H2AX. MDC1 must be recruited to γH2AX before MRN can bind in the outer focus. In a final step, ATM also binds to recruited MDC1. For clarity, only the nucleosomes that contain H2AX are depicted.

In order to test and validate the hypothesis of the two different binding modes of NBS1 being responsible for the observed changes in the recruitment dynamics with LET, we developed a numerical model for the early DNA damage response. It allows us to combine our individual experimental results over the whole range of LETs. It is based on a minimal subset of the known interactions between damage response proteins and DNA. Figure 10 gives an overview of the reactions that are found to be essential and included in our final model. Based on the experimental findings we included two qualitatively distinct interactions by which NBS1 (as MRN complex) binds to the DSB focus. The first reaction taken into account is the reversible binding of MRN directly to the DSB (inner focus binding). Our model does not contain any initial processing of the DSBs, but assumes that DSBs are ready for binding MRN. MRN bound to the DSB catalyzes the activation of ATM through auto-phosphorylation [14]. This is modeled as free ATM binding to MRN in the inner focus and subsequently dissociating as activated ATM. Active ATM will then phosphorylate H2AX to γH2AX. MDC1 binds directly to γH2AX [20] and MRN recruitment in the larger vicinity (outer focus binding) of the DSB is MDC1-dependent [21], [34], [35], [44]. It is known that ATM is retained at DSBs through interaction with MDC1 and that phosphorylation of ATM plays an important role here [14].

These protein interactions are translated into a system of ordinary differential equations that is then solved numerically. The results represent the protein concentration dynamics in the fixed volume around damage foci.

We reduced the number of model parameters by using dissociation rate constants determined in the FRAP measurements. All other model parameters were determined such that they resulted in the best fit to the recruitment curves for all LET values for NBS1, ATM, and MDC1 at the same time. It is important to notice, that a single model parameter set is used to fit all experimental data sets, with the only difference between individual simulations being the DSB input value, which scales linearly with LET [45].

We found that the best results are obtained when phosphorylated ATM is allowed to bind to MRN in the inner focus and, independent of the presence of MRN, to MDC1 recruited in the outer focus. The inclusion of further ATM interactions known from the literature, such as autophosphorylation of free ATM (inspired by [46]) or phosphorylation of H2AX by ATM bound in the outer focus (see [47]) did not improve the result. The inclusion of dephosphorylation reactions for γH2AX and active ATM as well as dissociation reactions for ATM at the outer focus did not change the simulation results significantly. Because we are interested in a minimal model of protein recruitment, these reactions were left out for the final calculations. Nevertheless, we want to emphasize that this does not mean that these processes do not occur. The fact that ATM dissociation does not affect our simulation results is due to the high concentration of ATM in the cell, leading to most ATM binding sites being occupied even when there is a high turnover of bound ATM.

**Figure 11:**
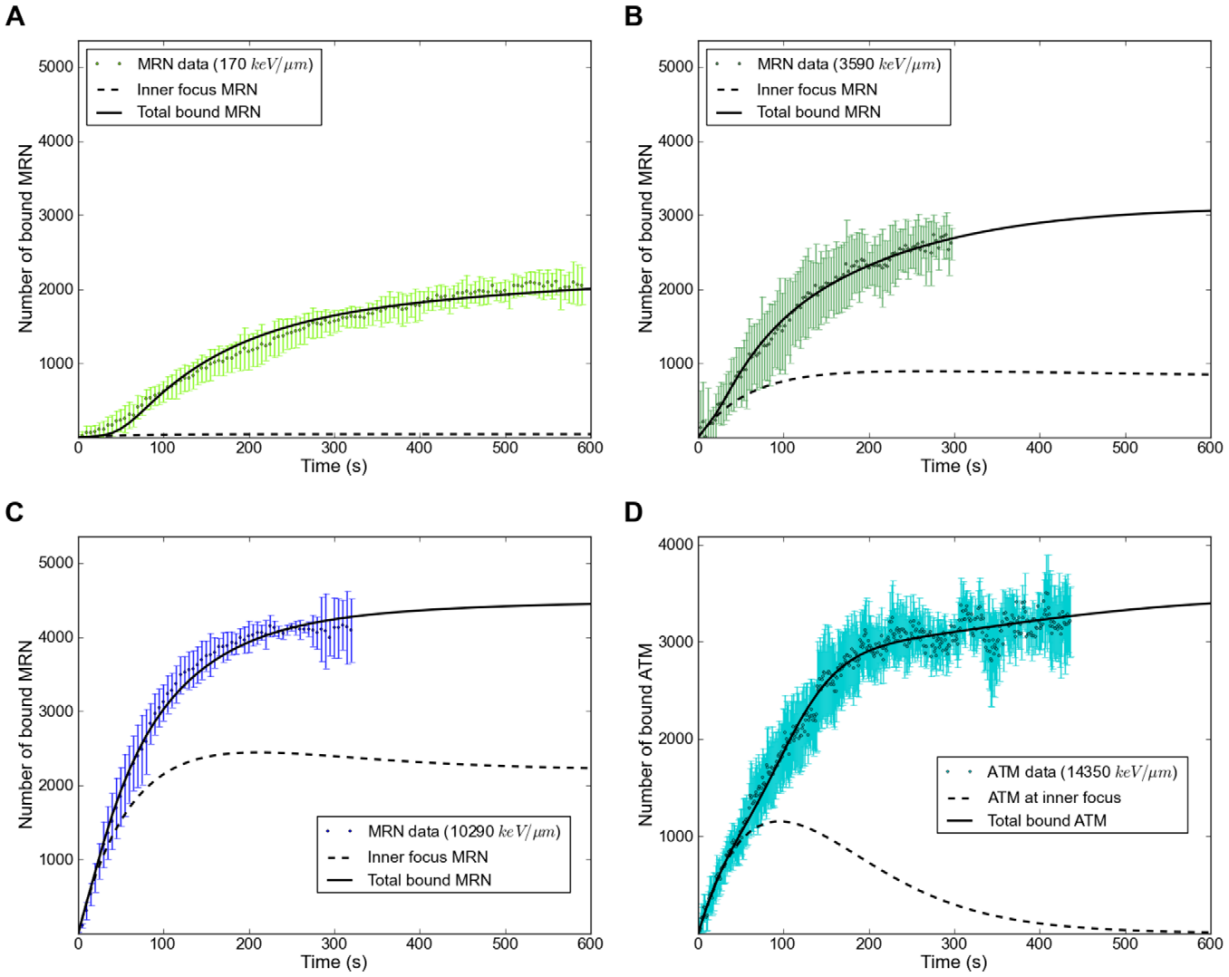
Comparison of NBS1 and ATM recruitment data with model results. A–C: NBS1 data and NBS1 signal calculated from the recruitment model for LETs of 170 keV/µm, 3590 keV/µm and 10290 keV/µm. Dashed lines indicate the NBS1 signal contribution of MRN recruited to the inner focus (MRN_i), whereas solid lines indicate total recruited NBS1 signal. D: ATM recruitment data and model for an LET of 14350 keV/µm. Dashed line indicates ATM bound at the inner focus, solid line indicates total recruited ATM. The concentration of H2AX in the focus, which limits binding sites for MRN and ATM in the outer focus, has a value of 3500. Additional figures for all of our recruitment data can be found in the supplementary material.

Figure 11 shows the resulting fit with the minimal model for three representative MRN data sets, as well as the ATM data set. In particular, the model reproduces successfully the increase in recruitment. The model results show that binding at the inner focus contributes significantly to the MRN concentration at high LET (Figure 11B and C), while it is almost negligible at low LET values (Figure 11A). This is due to the fact that the number of inner MRN binding sites increases linearly with LET, while the number of binding sites in the outer focus remains constant. The inner focus dynamics, whose contribution increases with LET, is faster than the outer focus dynamics. Consequently, the faster saturation for higher LETs reflects a shift in the shape of the recruitment curve from that of the outer focus to that of the inner focus.

**Figure 12:**
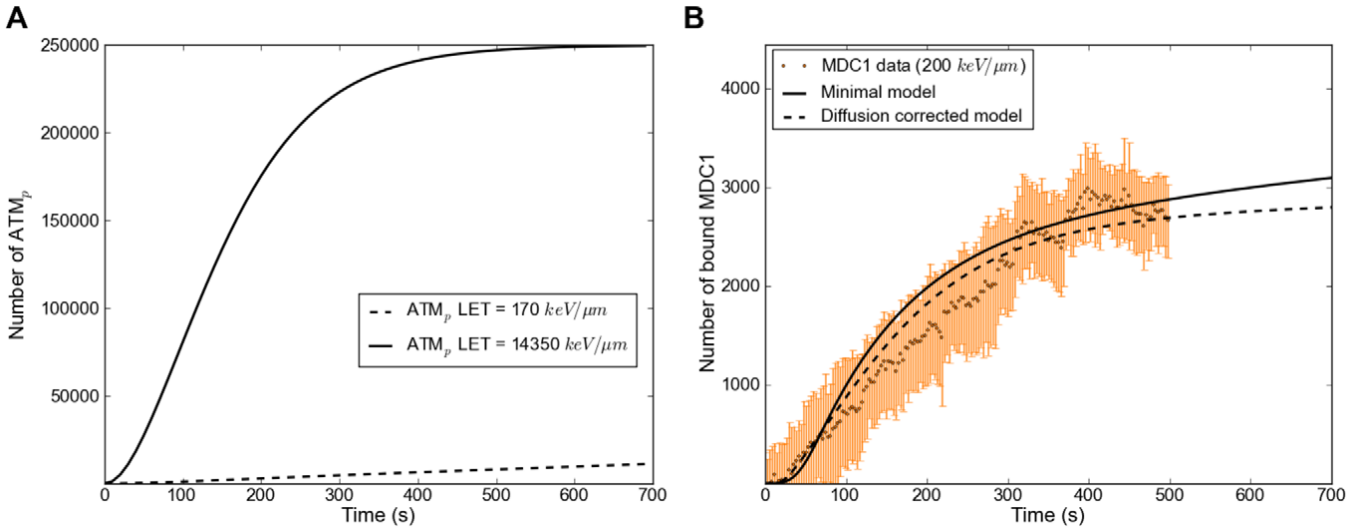
Active ATM in the model and comparison MDC1 model/experiment. A: Activation of ATM in the model for an LET of 170 keV/µm and of 14350 keV/µm. The high LET curve goes into saturation as all of the available ATM is activated. It has to be noted that the absolute maximum value for ATM is a relative value that represents the effective concentration of ATM (due to its fast diffusion throughout the nucleus). B: MDC1 data set for an LET of 200 keV/µm and the corresponding simulation results (solid curve). In this particular calculation the steady state concentrations for MDC1 are not reached in the first 700 s. For larger times, the total number of recruited MDC1 saturates at a value of 3500. The fit at low LET can be considerably improved by taking into account the slow diffusion of MDC1. When the amount of available MDC1 in the simulation is made to increase as (4Dt)^1/2^, as would be the case for diffusion in a cylindrical geometry, the dashed curve is obtained.

For very high LETs (solid line in figure 12 A), our model shows that all available ATM is activated within ten minutes, whereas for lower LETs (dotted line in figure 12 A) a state in which all ATM is activated is never reached during the course of a simulation. Since ATM binds to MRN in the inner focus during activation in our model, this behavior also influences the MRN recruitment curves. In the high LET simulation of figure 11C, there is a slight decrease in the amount of MRN at the inner focus about 200 s into the simulation. The reason for this is that binding of ATM to the inner focus impedes the dissociation of MRN, so that the MRN concentration in the inner focus is increased when there is a high level of ATM activation. Once all ATM is activated, the impediment disappears and the MRN in the inner focus level drops again. For low LETs (figure 11A and B), ATM activation happens too slow for this effect to appear.

Because all ATM is activated fast after high-LET irradiation the number of ATM bound at the inner focus decreases (Figure 11D dashed line). This effect also leads to a bend in the ATM recruitment curve (Figure 11D) around the time 300 s, where the steady increase in recruitment in the outer focus is counteracted by the decrease in the inner focus. The curve then continues on to saturate at time 600 s, when almost all binding sites for ATM in the outer focus are occupied.

Due to the relatively slow diffusion of MDC1only general agreement could be achieved between the model and the MDC1 recruitment data. When comparing our MDC1 data at low LET to MDC1 model results (Figure 12B), we see that the experimental curve has a constant slope that remains below the simulation result between 100 s and 300 s. This is consistent with a situation in which the MDC1 concentration is locally decreased because the diffusive influx of MDC1 becomes rate limiting for the recruitment reaction. To test this hypothesis, we modified our model so that the total amount of available MDC1 increases proportionally to (4Dt)^1/2^ (using D_eff_ = 0.029 µm^2^/s obtained through FRAP), which is the scaling behavior of diffusive motion in a cylindrically symmetric system. The result of this modified model (dashed curve in Figure 12B) shows improved agreement with the MDC1 recruitment data at low LET.

## Discussion

In this study we combined live cell imaging techniques and took advantage of charged particle irradiation to gain insights into the recruitment and binding kinetics of early damage response proteins at increasing lesions clustering.

As groundwork for the evaluation of the binding properties of NBS1 and MDC1, we analyzed the general protein mobility in the nuclear environment of untreated U2OS cells The diffusion coefficient of 12 µm^2^/s for pure GFP is in good agreement with published diffusion coefficients ranging from about 5 to 90 µm^2^/s [43], [48]–[56]. Based on these results the expected diffusion coefficients D_calc_ for the GFP-conjugated NBS1 and MDC1 proteins could be assessed. However, the experimentally determined effective diffusion constants D_eff_ were much lower than the calculated values. The estimation of the GFP-derived D_calc_ values are based on the assumptions that proteins have the same density and are spherically shaped. Consequently, more open protein structures would promote slower diffusion, but it seems unlikely that a deviation from the spherical shape could lead to the observed drastic decrease in effective diffusion. Additionally, the proteins could aggregate in larger complexes leading to a higher mass. This is described for NBS1, being part of the MRN complex and also for MDC1, interacting with NBS1 (for further protein interactions of NBS1 and MDC1 see reviews for example from [19], [57], [58]). However, because of the cube root relation between mass and diffusion coefficient, the complexes would need to be very large, in the order of 10^6^ kDa to 10^9^ kDa, to reach the measured D_eff_ values. Preformation of the MRN-complex consisting of one or even two RAD50, NBS1 and MRE11 molecules would not change the diffusion values dramatically. Another reason for slower NBS1 and MDC1 protein diffusion could be a mechanism of sliding along the chromatin for these proteins leading to the absence of 3-dimensional diffusion as described for some proteins like the repair proteins Rad51 and Msh2-Msh6 and transcription factor RNAP [59]–[62]. This behavior is restricted to proteins with very specific functions and there is to our knowledge no indication from literature data that this is the case for NBS1 or MDC1. Consequently the most probable explanation for the discrepancies between D_calc_ and D_eff_ is a temporary binding of the proteins to immobile structures in the nucleus, e.g. non-damaged DNA, either directly or via other interaction partners. This putative binding was found to be more pronounced for MDC1 than for NBS1 explaining also the observed small deviation in the effective diffusion fit (Figure 4B). The true nature of these binding sites in non-irradiated cells is a matter of future studies. In agreement with our data Lukas and coworkers also described a higher mobility for NBS1 than for MDC1 [44], [63]. However, they published higher D_eff_ values of 2.53 µm^2^/s ±0.3 µm^2^/s for NBS1 and 2.08 µm^2^/s ±0.29 µm^2^/s for MDC1, which may be due to different experimental conditions [44]. In general, published diffusion coefficients for nuclear repair proteins involved in different pathways range from 0.35 µm^2^/s to 28 µm^2^/s [49], [64], [65]. Thus our results are at the lower limit and below indicating that stronger binding to immobile structures might not be a general phenomenon.

With this groundwork we were able to analyze protein mobility after ionizing irradiation. We performed FRAP measurements in cells containing DSBs after protein accumulation had reached steady state.

Taking advantage of the locally confined dose deposition and the modulation of the lesion density using charged particle irradiation, we provide here *in vivo* support that NBS1 is binding in two different fractions at DSBs as suggested in previous experiments using laser micro- and X-ray irradiation [21], [33]–[35], [44]. Most probably, NBS1 can either bind more directly to DSBs (inner focus) or to the surrounding chromatin via MDC1 and γH2AX (outer focus) as described previously [36]. These two binding modes might also represent NBS1 binding in complexes of different composition. The binding in the outer focus via MDC1 can be suppressed by CK2 inhibition, which leads to a dephosphorylation of MDC1. Phosphorylation at the N terminus SDTD repeats is a prerequisite for binding to the MRN complex [21], [33]–[35]. After CK2 inhibition by TBB, only the inner focus binding of NBS1 to the DSBs remains, which can be demonstrated by the formation of small radiation induced NBS1 dots, clearly discriminable from the normal IRIF after immunostaining for NBS1 (Figure 8B). Taking advantage of that effect in our live cell approach, we could obtain the binding constants for NBS1 binding directly to DSBs by fitting the FRAP measurements after CK2 inhibition. The number of NBS1 molecules in the inner focus is expected to increase with LET, as the number of DSBs also increases. The number of NBS1 molecules bound via MDC1 saturates at a certain damage density, when all surrounding H2AX molecules of the damaged chromatin domain are phosphorylated. Thus this hypothesis predicts that with increasing LET the fraction of NBS1 molecules directly bound to DSBs increases and the binding constants should approach those of NBS1 binding in the inner focus. Strikingly, this behavior could be observed in our experiments (Figure 8A) as for very high LETs the obtained k_off_ values approached that after CK2 inhibition.

The dynamics of NBS1 proteins in non-irradiated areas of the cell nucleus was found to be identical to that in untreated cells, indicating no irradiation-dependent additional binding in regions where no dose was deposited. In contrast, MDC1 unexpectedly showed a clearly reduced mobility in the whole nucleus after irradiation with very high LET. The corresponding curves could no longer be described with the effective diffusion model suggesting pronounced binding. Thus the diffusion-reaction model with global binding was applied and the data could be described reasonably well, yielding the corresponding binding constants.

For both types of MDC1 binding, the pan-nuclear one after high LET irradiation as well as locally at DSBs after low LET irradiation, we observed similar k_off_ values, indicating the same molecular mechanism, namely binding to γH2AX. Pan nuclear MDC1 binding after high LET irradiation was investigated in more detail in our group. The results are beyond the scope of this article and a matter of a separate publication by Meyer and coworkers [66]. The higher k*_on_ values for local damage compared to global chromatin binding indicate higher phosphorylation densities around DSBs and thus more free binding sites for MDC1.

The basal question we addressed in this study was aimed at elucidating mechanistic differences influencing the accumulation dynamics of repair protein at higher DSB densities instantly after irradiation. The recruitment kinetics in general strongly depends on the velocity of binding site generation comprising, besides lesion induction itself, chemical modifications or the recruitment of interacting proteins. Thus the kinetics is a consequence of the proteins' position in the reaction cascade. Strikingly NBS1, ATM and MDC1 all showed fast foci formation, consistent with their early function in the DNA damage response. In this context it is interesting to see that the rather low diffusion rate measured for MDC1 in undamaged cells is still not rate limiting for the recruitment to damage sites after irradiation. However we presume that the pan-nuclear immobilization after high LET irradiation could influence recruitment dynamics at a subsequent insult.

In contrast to these early responding proteins, 53BP1 foci were not detectable up to 100 s, even after high LET irradiation (Figure S2). This clearly indicates upstream processing steps prior to 53BP1 binding. According to previously reported results these may include additional RNF8, UBC13, RNF168, HERC2 protein binding, ubiquitination and degradation by JMJD2A after the accumulation of MDC1 ([67] and review see e.g. [68], [69]).

These results of NBS1, MDC1 and 53BP1 accumulation are in good agreement with studies by Lukas et al. and Bekker-Jensen et al. [44], [70] using UV Laser micro-irradiation to generate local DNA damage. However even after sensitization, UV irradiation might cause different types of DNA damage compared to ionizing radiation and the applied biological dose equivalent cannot be determined precisely [39].

Utilizing charged particle irradiation with different LET we found that the velocity of protein accumulation depends not only on the protein itself [71] but also largely on the radiation quality or damage density. This is in agreement with recruitment kinetics recently reported for lower LETs [26], [72] and could potentially impact on further repair as pathway choice by influencing the timing of subsequent steps.

We assumed that the increase of recruitment rates of NBS1 with increasing LET is due to the interplay between the inner and outer focus binding. To test this hypothesis and to bring the results into a quantitative context, we developed a mathematical model, consisting of a set of ordinary differential equations that describe those protein interactions that we considered and prove to be absolutely necessary to reproduce the data. Such models have been the standard approach for damage response protein dynamics modeling in recent years [46], [73], [74]. We want to recall that there are several early proteins, such as the NHEJ proteins DNA-PKcs and Ku70/80, which we neither measured nor included in our model. We restricted our investigations to the pathway-independent damage response and thus the MRN-ATM-H2AX-MDC1 subunit of the response network, assuming that other proteins do not significantly affect the interactions included (For pathway dependent model see for example [75]). The dissociation rates k_off_ of MRN and MDC1 obtained by our FRAP measurements were put into the model. The number of binding sites in the inner focus was taken to increase linearly with the number of DSBs. Addition of a quadratic term taking the formation of additional DSBs by interacting SSBs into account will not change the results significantly. The number of binding sites in the outer focus was assumed to be constant and amounting to a few thousand.

Our modeling results show that for the lowest LET data sets binding of MRN at the inner focus is negligible compared to the outer focus, while it becomes the dominating binding type for the highest LET values contributing to nearly 60% for uranium. This leads to NBS1 accumulation becoming faster with increasing LET and thus explains the LET dependency of NBS1 recruitment data.

Comparing the activation of ATM for different LET values (Figure 12A), we see that while only a small fraction of ATM is activated in the first minutes of low-LET irradiation, all ATM is activated for high LET values. Consequently, outer focus binding sites for MRN become available faster in the high LET case. The difference in the contribution of the outer foci to the recruitment curves for MRN at the lowest and highest LET we investigated is only a delay of the order of some tens of seconds. This is not surprising, as the lowest LET value (170 keV/µm) still corresponds to 28 DSBs, meaning that even at our lowest LET we encounter the cellular response to what would be a large number of DSBs in a natural environment. In agreement with this idea, at even lower LETs a slower MDC1 recruitment was recently reported by Hable et al [26] for proton irradiation (LET = 2,6 keV/µm). Overall our mathematical model puts the concept of enhanced ATM activation with increasing LET on a solid foundation.

Our model does not contain a nucleus-wide phosphorylation of H2AX [66] and subsequent binding of MDC1 for high LET values. Therefore for these LET values (in Figure S4) our experimental MDC1 recruitment data saturates earlier than the model predicts. A largely reduced free MDC1 population due to MDC1 binding at γH2AX in the entire nucleus could cause such premature saturation. A preliminary modification of our model to include nucleus-wide interactions showed improved agreement with high-LET MDC1 recruitment data.

Overall our results prove charged particle irradiation as a powerful tool to study the mechanisms of the cellular DNA damage response. We have shown the influence of high damage densities on the dynamics of early repair proteins both within and distant from damaged chromatin. We find that clustered damage induced by densely ionizing charged particles can lead to differences in recruitment kinetics and binding modalities of repair factors without the need to infer on different molecular mechanisms. Nonetheless these LET dependent changes in dynamics might influence the interplay of subsequent repair factors, and thus impact on damage signaling and repair.

## Materials and Methods

### Cell culture

All cells were cultured in 75 cm^2^ or 25 cm^2^ culture flasks (BD Bioscience, Le Pont De Claix, France) by 37°C, 95% humidity and 5% CO_2_. Human osteosarcoma cells (U2OS) (ATCC, Wesel, Germany) and U2OS-NBS1-GFP, U2OS-MDC1-GFP and U2OS-53BP1-GFP (all kindly provided by Dr. Claudia Lukas Danish Cancer Society, Copenhagen, Denmark) cells were cultured in DMEM medium (Biochrom AG, Berlin, Germany) and 10% fetal calf serum. U2OS-derived cell lines stably expressing NBS1-GFP, MDC1-GFP and 53BP1-GFP were described and characterized previously showing the functionality of the GFP-constructs [44], [70], [76].

### Cell Irradiation

Charged particle irradiation was performed at GSI accelerator for heavy ions as described earlier [32], [77].

Alternatively, cells were irradiated with 250 kV X-rays at a dose rate of 2–3 Gy/min. The exit window consisted of 7 mm beryllium and additional filters of 1 mm copper 1 mm aluminum. The adopted LET was 1 keV/µm.

### Protein recruitment experiments

90,000 cells were seeded 1 day or 60,000 cells 2 days before the experiment on round disks (18 mm ø) of either 40 µm thick polycarbonate films (Goodfellow GmbH, Bad Nauheim, Germany), 25 µm thick lumox film 25 (In Vitro Systems & Services, Göttingen, Germany) or on glass cover slips (Menzel GmbH & Co KG, Braunschweig, Germany). Analysis of cell cycle distribution by flow cytometry revealed that under these conditions at least 50% of cells are in G1 phase.

The beamline microscope setup was described earlier [27], [78]. Fluorescence was excited with the monochromator Polychrome V (TILL Photonics GmbH, Gräfeling, Germany). Image acquisition was done with an EM-CCD camera type DU-888 or DV-887 (Andor Technology, Belfast, Ireland) and the corresponding AndorIQ software.

### FRAP experiments

500,000 or 250,000 cells were seeded on 40 mm diameter glass cover slips (Menzel GmbH & Co KG, Braunschweig, Germany) 1 or 2 days before the experiment, respectively. Samples were mounted in Focht Chamber System 2 (Bioptechs Inc, Butler, USA).

A Leica (Leica Microsystems GmbH, Wetzlar, Germany) DM LA microscope with HCX PL Fluotar 100x 1.3 oil immersion lens and CTR MIC control unit was used. A 100 mW 473 nm diode laser type DPL 473-OEM (Rapp Opto Electronics, Hamburg, Germany) was coupled in the microscope by a modified Leica AS LMD module and dicroic mirror Q480 LP (Chroma Technology Corporation, Bellows Falls, USA). The bleach laser was controlled by the Leica Laser Microdissection System LMD version 4.4.0.0. The monochromator and cameras described in the protein recruitment experiments section, were also used here. Measurements were performed controlling the bleach spot characteristics according to the work of McNally [30]. The FWHM of the bleaching spot was (3.0±0.2) µm.

### Immunocytochemistry

U2OS cells were fixed for 15 min in 2 % paraformaldehyde solution and permeabilized for 10 min with 0.5% Triton X-100 as described [37]. After 2 times washing with PBS cells were blocked in 0.5% BSA in PBS. Immunostaining was done as described before [79]. Rabbit NBS1 p95 (ab23996) (1∶300, Abcam, Cambridge, UK) and sheep MDC1 (1∶400, kindly provided by Dr. Nuri Gueven, The Queensland Institute of Medical Research, Brisbane, Australia) antibody solutions in 0.4% BSA in PBS were used. Secondary antibodies were Alexa goat 488 anti-rabbit and 568 anti-sheep (all Invitrogen). DNA was counterstained with a 4′,6-diamidino-2-phenylindole (DAPI) solution of 1 µg/ml in PBS for 15 min. Samples were mounted in Vectrashield Mounting Medium (Vector Laboratories, Burlingame, U.S.A.).

CK2 inhibition was performed with TBB (4,5,6,7-Tetrabromo-2-azabenzimidazole). Cells were incubated in a solution of 300 µM TBB 5 h before irradiation and afterwards.

IF images were acquired using the Andor (Belfast, UK) Revolution System equipped with a Nikon (Düsseldorf, Germany) TiE microscope, a Yokogawa (Tokyo, Japan) CSU-X1 spinning disk confocal scanner and a Nikon 100x PlanApoVC 1.4 NA oil immersion lens.

### Image analysis

Image analysis was performed with the software ImageJ (http://rsweb.nih.gov/ij/). Cell motion during acquisition was compensated with the StackReg plugin (Philippe Thevenaz, Lausanne, Switzerland) or with the object stabilizer in the Huygens Essential software (Hilversum, Netherlands).

The measurements were double normalized to the prebleach intensity and to signal loss during image acquisition according to [80].

The Laplace transforms of the diffusion reaction models solution described by Sprague and coworkers [40], [43] were numerically inverted in Wolfram Mathematica with the Numerical Inversion package Version 1.0 (Arnaud Mallet, University of Mauritius) using the Stehfest method.

FRAP curves were fitted with the nonlinear regression package (John M. Novak and E. C. Martin) for the Wolfram Mathematica software. To find the local minimum, the start values for k*_on_ and k_off_ were permuted in 10 times steps from 10^−6^ to 10^6^.

### Mathematical model

All simulations were performed using the netdyn python package for chemical reaction computing, which was developed by one of the authors and is available online [Löb D. netdyn – a chemical reaction network dynamics package. www.danielloeb.eu/netdyn.html]. The package automatically generates differential equations from the set of chemical reactions, which are then solved using the Runge-Kutta Cash-Karp method [81].

Details and equations of the model can be found in the Supporting Information S1.

### Parameter Estimation

For the optimization of our model, we used a series of twelve recruitment data sets for NBS1, three data sets for MDC1 and one ATM recruitment data set. In each optimization step, for each NBS1 and ATM data set a calculation is performed, with all calculations using identical parameters for reaction rates, total concentrations and data set scaling. The only parameter that is changed between simulation runs is the number of DSBs, which is obtained from the LET value of each data set. Least squares between data points and the corresponding function values are summed up over all calculations to serve as the optimization measure.

All optimizations were done using the Nelder-Mead downhill simplex algorithm [82] provided by the python scipy package. The set of parameters that resulted from our optimization is contained in the Supporting Information S1.

## Supporting Information

Figure S1 **All NBS1 recruitment data sets with the corresponding model calculations.** Protein concentrations and rate constants were identical for all model calculations. Only the number of DSBs was set to a different value (calculated from LET) for each simulation. Curves B, I and L are shown in the main text. The curve color codes for LET.
(TIF)

Figure S2 **53BP1 protein accumulation at damaged DNA sites after C- and Au-ion irradiation.** Relative fluorescence intensity of GFP tagged 53BP1 accumulating at DNA damage. Curves are normalized to 0 before irradiation and to 1 for the plateau value. The kinetics is not LET dependent.
(TIF)

Figure S3 **FRAP curve of pure GFP in the nucleus of U2OS cells.** Human osteosarcoma cells (U2OS) were cultured as described in the Materials and Methods section. GFPmut1 plasmid [83] was kindly provided by S. Scott (The Queensland Institute of Medical Research, Brisbane, Australia) and transfected with the Amaxa Nucleofector I. Data were fitted with the diffusion model described by Soumpasis [29].
(TIF)

Figure S4 **All MDC1 and ATM recruitment data sets with the corresponding model calculation.** MDC1 data sets were not used in the model parameter optimization, so the absolute values shown here are chosen for best comparability. Figures A and D are shown in the main text.
(TIF)

Table S1 **Effective association-** (k*_on_) **and dissociation** (**k_off_**) **constants of NBS1.**
(DOC)

File S1 **Mathematical model.**
(DOC)
