# Supplementary figures and images for: Spatiotemporal Dynamics of Early DNA Damage Response Proteins on Complex DNA Lesions
Source: PLoS One. 2013 Feb 26;8(2):e57953. doi: 10.1371/journal.pone.0057953 (PMC3582506; doi:10.1371/journal.pone.0057953)

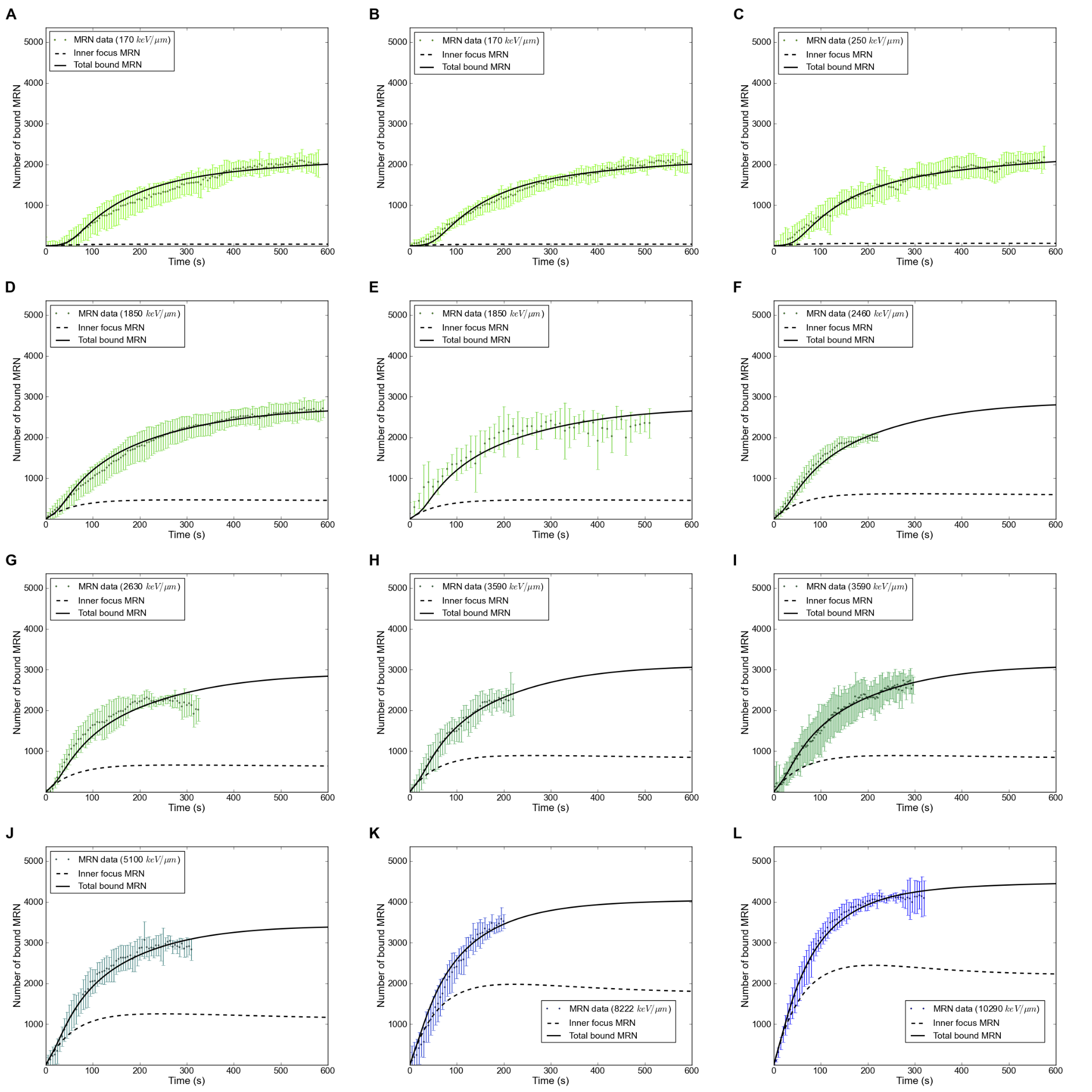

Supplement: Figure S1 — All NBS1 recruitment data sets with the corresponding model calculations. Protein concentrations and rate constants were identical for all model calculations. Only the number of DSBs was set to a different value (calculated from LET) for each simulation. Curves B, I and L are shown in the main text. The curve color codes for LET. (TIF) [file pone.0057953.s001.tif]

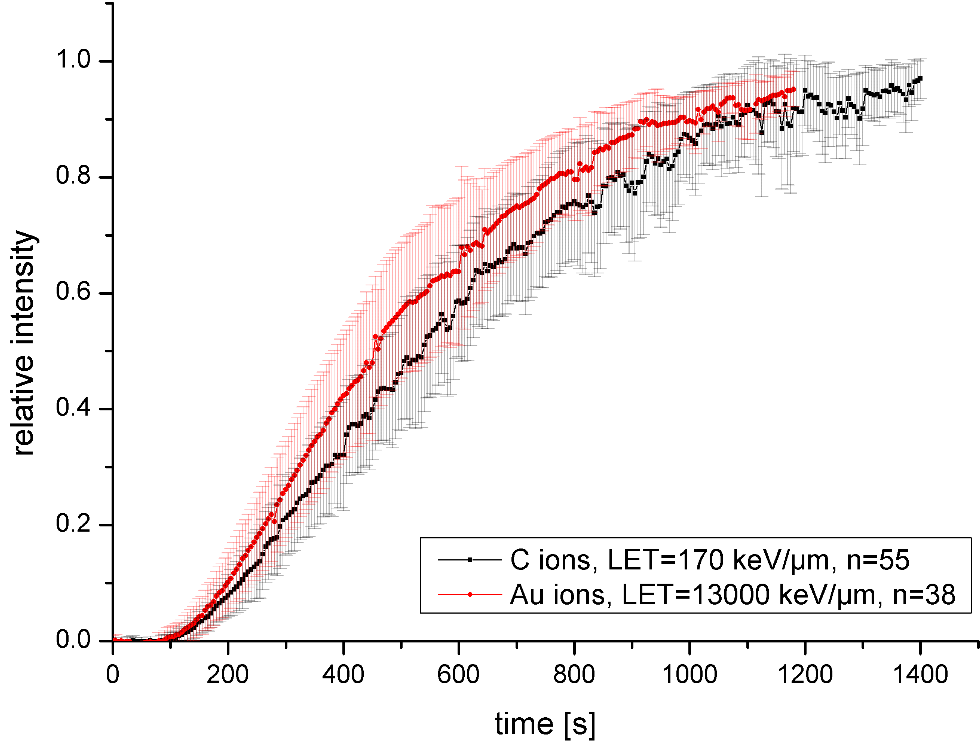

Supplement: Figure S2 — 53BP1 protein accumulation at damaged DNA sites after C- and Au-ion irradiation. Relative fluorescence intensity of GFP tagged 53BP1 accumulating at DNA damage. Curves are normalized to 0 before irradiation and to 1 for the plateau value. The kinetics is not LET dependent. (TIF) [file pone.0057953.s002.tif]

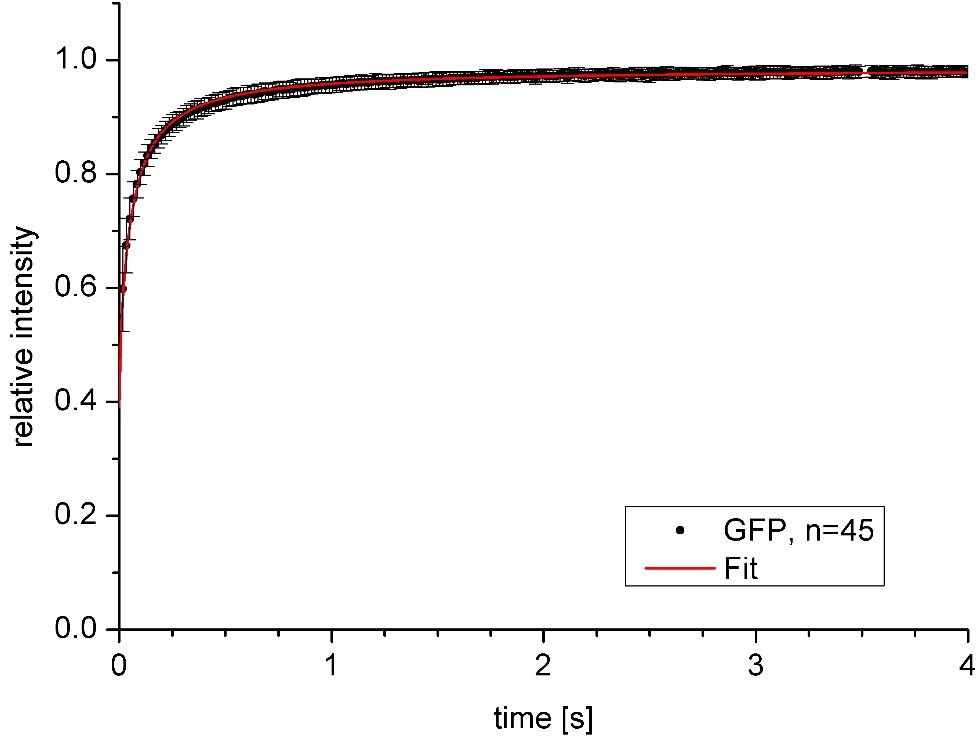

Supplement: Figure S3 — FRAP curve of pure GFP in the nucleus of U2OS cells. Human osteosarcoma cells (U2OS) were cultured as described in the Materials and Methods section. GFPmut1 plasmid [83] was kindly provided by S. Scott (The Queensland Institute of Medical Research, Brisbane, Australia) and transfected with the Amaxa Nucleofector I. Data were fitted with the diffusion model described by Soumpasis [29]. (TIF) [file pone.0057953.s003.tif]

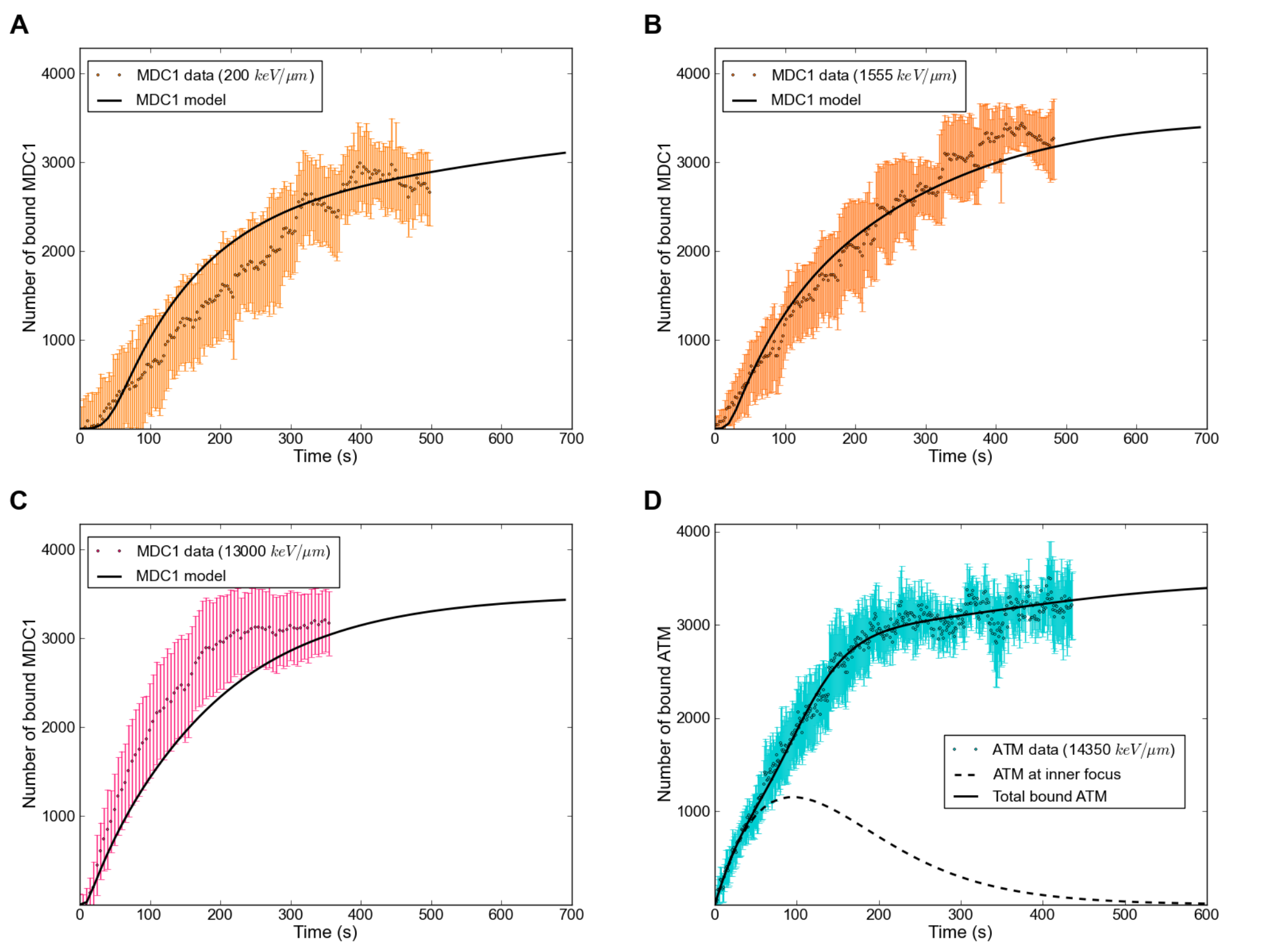

Supplement: Figure S4 — All MDC1 and ATM recruitment data sets with the corresponding model calculation. MDC1 data sets were not used in the model parameter optimization, so the absolute values shown here are chosen for best comparability. Figures A and D are shown in the main text. (TIF) [file pone.0057953.s004.tif]
