# Supplementary material for: Spatiotemporal Dynamics of Early DNA Damage Response Proteins on Complex DNA Lesions
Source: PLoS One. 2013 Feb 26;8(2):e57953. doi: 10.1371/journal.pone.0057953 (PMC3582506; doi:10.1371/journal.pone.0057953)
Supplement: Table S1 — Effective association- (k*on) and dissociation (koff) constants of NBS1. (DOC) [file pone.0057953.s005.doc]

**Table S1:** Effective association- (k*_on_) and dissociation (k_off_) constants from fitting the FRAP curves of NBS1 binding at damaged DNA (Figure 6 and 7) with the mathematical radial diffusion-reaction model described by Sprague and coworker [Sprague BL et al. (2006) Biophysical Journal 91: 1169–1191].

| **Irradiation** | **LET [keV/µm]** | **k*_on_ [1/s]** | **k_off_ [1/s]** |
| --- | --- | --- | --- |
| X-ray | 1 | 0.372 ± 0.024 | 0.047 ± 0.002 |
| C-ions | 170 | 0.089 ± 0.004 | 0.026 ± 0.001 |
| Ar-ions  + CK2 inhibition | 1550 | 0.164 ± 0.008  0.016 ± 0.004 | 0.023 ± 0.001  0.007 ± 0.001 |
| Ni-ions | 3430 | 0.243 ± 0.008 | 0.030 ± 0.001 |
| Xe-ions | 8655 | 0.071 ± 0.005 | 0.010 ± 0.001 |
| U-ions | 14350 | 0.077 ± 0.004 | 0.011 ± 0.001 |
| U-ions  + CK2 inhibition | 15000 | 0.0257 ± 0.0013  0.0280 ± 0.0015 | 0.0054 ± 0.0002  0.0040 ± 0.0001 |
