# Supplementary material for: Spatiotemporal Dynamics of Early DNA Damage Response Proteins on Complex DNA Lesions
Source: PLoS One. 2013 Feb 26;8(2):e57953. doi: 10.1371/journal.pone.0057953 (PMC3582506; doi:10.1371/journal.pone.0057953)
Supplement: File S1 — Mathematical model. (DOC) [file pone.0057953.s006.doc]

**Supporting Information S1**

**Mathematical model**

The chemical reactions of our minimal model are:

MRN + DSBs ⇌ MRNi (1)

ATM + MRNI → AMRNi (2)

AMRNi → ATMp + MRNi (3)

H2AX + ATMp → γH2AX + ATMp (4)

MDC1 + γH2AX ⇌ MγH2AX (5)

MRN + MγH2AX ⇌ MMγH2AX (6)

MγH2AX + ATMp → AMγH2AX (7)

MMγH2AX + ATMp → AMMγH2AX (8)

MRN + AMγH2AX ⇌ AMMγH2AX (9)

The compound species are:

DSBs: Double strand ends that are available for direct binding by MRN. Since each DSB consists of two double strand ends, the number of DSB s is double that of double strand breaks.

MRNi: MRN bound to a double strand end.

AMRNi: ATM bound to an MRN i.

ATMp: Activated ATM.

MγH2AX: MDC1 bound to γH2AX in the surrounding chromatin.

MMγH2AX: MRN bound to MDC1 in the surrounding chromatin.

AMγH2AX: AT M bound to recruited MDC1.

AMMγH2AX: AT M bound to recruited MDC1 that also binds MRN.

Equation (9) is added to keep binding of ATM and MRN fully independent. There are no new reaction parameters introduced with equations (8) and (9) because they use the same reaction rate parameters as equations (7) and (6).

The additional ATM reactions mentioned in the discussion that were tested but were not included in the minimal model are:

ATMp + ATM → 2ATMp (10)

H2AX + AMγH2AX → γH2AX + AMγH2AX (11)

H2AX + AMMγH2AX → γH2AX + AMMγH2AX (12)

In order to keep the number of fit parameters as small as possible, we used the experimentally obtained rate constants as input. In particular, we used the dissociation rates for MRN in the inner and outer focus as well as for MDC1 from the results of our FRAP measurements. We also made sure that the k*_on_ values calculated by the model in the steady state are of the same order of magnitude as the values obtained in the FRAP measurements As the number of binding sites in the inner focus is proportional to the number of DSBs, for very low LETs it becomes negligible compared to the DSB-independent number of binding sites in the outer focus. We therefore used the NBS1 dissociation value for X-ray irradiation as the outer focus MRN dissociation rate: k_off MRN, o_ = 0.047 1/s. As mentioned above, inhibition of kinase CK2 inhibits binding of MRN at the outer focus. We therefore used the dissociation rate obtained in FRAP measurements with CK2 inhibition (see Figure 8A) k_off MRN, i_ = 0.007 1/s as the rate for dissociation of MRN in the inner focus. For the MDC1 dissociation rate we used the value k_off MDC1_ = 0.002 1/s that was determined in our FRAP measurements.

The number of DSBs per traversal was assumed to scale linearly with LET [Löbrich et al. (1994) Radiat Res 139: 142–151]. The linear parameter for the number of DSBs was determined from the ion fluence (in our experiments 3·10^6^ 1\cm^2^), the LET, and the assumption that there are on average 35 DSBs per Gray [Prise KM et al. (1998). IJRB 74: 173–184], resulting in 28 DSBs at an LET of 170 keV/mu.

Due to the slow diffusion of MDC1 we expected less agreement between the model and MDC1 recruitment data than between the model and other data sets. For this reason, we used the MDC1 data sets only to determine the time at which MDC1 recruitment is saturated and then added a term to the optimization measure that punishes saturation at a later time for the corresponding LET.

This is the entire set of parameters that was used in the calculations presented in the main text.

Optimized rate constants are (in 1/s):

| ATM + MRNi → AMRNi | 3.75473e-06 |
| --- | --- |
| AMRNi → ATMp + MRNi | 0.989102 |
| H2AX + ATMp → gH2AX + ATMp | 0.000159441 |
| MDC1 + gH2AX → MgH2AX | 3.62805e-08 |
| MgH2AX → MDC1 + gH2AX and MRN + AMgH2AX → AMMgH2AX | 6.64206e-07 |
| MgH2AX + ATMp → AMgH2AX and MMgH2AX + ATMp → AMMgH2AX | 3.18033e-07 |
| MRN + DSBs → MRNi | 1.01616e-07 |

Rate constants determined from experiment are:

| MgH2AX → MDC1 + gH2AX | 0.00425 |
| --- | --- |
| MRNi → MRN + DSBs | 0.007 |
| MMgH2AX → MRN + MgH2AX andAMMgH2AX → MRN + AMgH2AX | 0.047 |

Protein concentration values are:

| ATM0 | 221859 |
| --- | --- |
| MDC10 | 162208 |
| MRN0 | 129056 |
| H2AX0 in focus | 3363 |

Scaling parameters for the Nbs1 data sets, identified with the same letters as Figure S1:

| A | 2032 |
| --- | --- |
| B | 2149 |
| C | 2059 |
| D | 2667 |
| E | 2289 |
| F | 1963 |
| G | 2196 |
| H | 2164 |
| I | 2688 |
| J | 2850 |
| K | 3414 |
| L | 4030 |

The scaling parameter for the ATM data set was 3263.
